# Integrative Approach of Treating Early Undernutrition with an Enriched Black Corn Chip, Study on a Murine Model

**DOI:** 10.3390/nu16132001

**Published:** 2024-06-24

**Authors:** Mercedes-Victoria Urquiza-Martínez, Imelda M. Fabián-Avilés, Luz Torner, Hermelinda Servín-Campuzano, Mauricio González-Avilés

**Affiliations:** 1 Master in Engineering for the Energetic Sustainability, Universidad Intercultural Indígena de Michoacán, Campus Tzipekua, Pátzcuaro 61614, Mexico; lnmurquiza@live.com (M.-V.U.-M.); mire_yangl@hotmail.com (I.M.F.-A.); shermelinda@uiim.edu.mx (H.S.-C.); 2 Centro de Investigación Biomédica de Michoacán, Instituto Mexicano del Seguro Social, Morelia 58330, Mexico; luz_torner@yahoo.com

**Keywords:** physical activity, energy expenditure, black corn, post-weaned undernutrition mouse model, thermogenesis

## Abstract

Undernutrition (UN) increases child vulnerability to illness and mortality. Caused by a low amount and/or poor quality of food intake, it impacts physical, cognitive, and social development. Modern types of food consumption have given highly processed food a higher cultural value compared to minimally processed food. Objective: The objective of this study was to evaluate the effect on growth, metabolism, physical activity (PA), memory, inflammation, and toxicity of an enriched black corn chip (BC) made with endemic ingredients on post-weaned UN mice. Methods: A chip was made with a mixture of black corn, fava beans, amaranth, and nopal cactus. To probe the effects of UN, UN was induced in 3wo post-weaned male C57Bl/6j mice through a low-protein diet (LPD—50% of the regular requirement of protein) for 3w. Then, the BC was introduced to the animals’ diet (17%) for 5w; murinometric parameters were measured, as were postprandial glucose response, PA, and short-term memory. Histological analysis was conducted on the liver and kidneys to measure toxicity. Gene expression related to energy balance, thermogenesis, and inflammation was measured in adipose and hypothalamic tissues. Results: Treatment with the BC significantly improved mouse growth, even with a low protein intake, as evidenced by a significant increase in body weight, tail length, cerebral growth, memory improvement, physical activation, normalized energy expenditure (thermogenesis), and orexigenic peptides (AGRP and NPY). It decreased anorexigenic peptides (POMC), and there was no tissue toxicity. Conclusions: BC treatment, even with persistent low protein intake, is a promising strategy against UN, as it showed efficacy in correcting growth deficiency, cognitive impairment, and metabolic problems linked to treatment by adjusting energy expenditure, which led to the promotion of energy intake and regulation of thermogenesis, all by using low-cost, accessible, and endemic ingredients.

## 1. Introduction

Health problems linked to nutritional factors are the leading preventable causes of illness and death around the world [1]. Undernutrition (UN) is still one of the main epidemiologic health problems of international concern. UN affects nearly 200 million children under the age of 5 who present either height-for-age deficiency (stunting) or weight-for-height deficiency (wasting). These conditions are caused by a lower-than-recommended intake of energy, protein, or both, combined with micronutrient (vitamins and minerals) deficiency in the early years of life [2]. Growth increases nutrient demand, and nutrient deficiencies in the early stages of life are closely related to under-development and learning problems, downregulated immune response and cicatrization, higher risk of future metabolic alterations (obesity, diabetes, hypertension, and dyslipidemia, to mention some), and weight and length shortfall [3]. This problem affects communities and countries of low- and middle-income socioeconomic conditions [2].

Energy balance (regulation of energy to determine its storage or expenditure) is a critical factor in promoting adequate growth linked to general good health [4]. Energy balance is guarded by several factors, the most important being the melanocortin system orchestrated on the hypothalamic arcuate nucleus. Among its functions are the regulation of food intake and body weight [5]. The melanocortin system integrates the signals from Proopiomelanocortin (POMC) neurons, which promote satiety, and Neuropeptide Y (NPY) and Agouti-Related Protein (AgRP) neurons, which induce appetite [6]. This is accomplished by interpreting the peripheral nutrient hormones (such as leptin from adipose tissue) and peptides that pass through the blood–brain barrier (BBB). Dietary essential amino acids (leucine and tryptophan) acquired from protein intake and levels of circulating glucose and fatty acids determine energy balance. In the early stages of life, these signals condition the neurogenesis process and the formation of functional circuits of POMC neurons, reaching their highest functionality between postnatal days 28–35 in mice. So, there is a close interaction between perinatal nutrition and the architecture of the melanocortin system in regulating body weight, satiety–hunger signals, energy expenditure, and even growth [7].

These effects are closely controlled intracellularly by the mammalian Target of Rapamycin Complex 1 (mTORC1), a nutrient, mitogenic, stress, and energy-sensitive regulator. The controlled presence of ROS (Reactive Oxygen Species) on hypothalamic POMC neurons favors satiety linked to the function of leptin in this neurologic circuit [8]. However, sustained levels of ROS can cause oxidative stress and mTOR disruption, which is related to neurodegenerative and cardiovascular diseases, obesity, and other metabolic disorders [9,10]. Oxidative stress is caused by the low response of the antioxidant system of an organism. Physiologically, a certain amount of reactive oxygen and nitrogen species (ROS and RNS) are needed to maintain homeostasis, as in the example mentioned before. However, when levels exceed normality, ROS/RNS affect lipids from cell membranes (lipoperoxidation), proteins, or even DNA, compromising its correct function or causing inflammation or cell death (apoptosis) [11]. One of the antioxidant defense mechanisms of an organism is the endogenous presence of antioxidant enzymes, like catalase, superoxide dismutase and glutathione peroxidase, and another one is the dietary intake of low-weight antioxidant molecules like vitamin E (tocopherol), vitamin C (ascorbic acid), carotenoids, copper (Cu), selenium (Se), zinc (Zn), and phenolic compounds [12]. So, dietary factors are crucial for downregulating the deleterious effects of ROS/RNS. The relation between UN and oxidative stress establishes that children with stunted growth show significantly reduced antioxidant enzyme levels (catalase, superoxide dismutase, and glutathione peroxidase), plasma Cu, Zn, and vitamin C, as well as total plasma proteins [13].

Functional food sources, natural or designed, are gaining interest in the scientific community given their effectiveness against multiple non-communicable diseases and their nutritional function; they have low to no side effects and are affordable and accessible. In this sense, corn, in general, is considered the third most consumed cereal after rice and wheat; it has a consumption trend of 50 kg/capita/year in countries from Africa and Latin America (LA), which are the most vulnerable sites for children according to the United Nations [14]. Corn is a nutritious and versatile crop product that belongs to the whole grain food group; studies like ELANS highlight the importance of its consumption in reducing the health system burden, making the promotion of corn consumption a cost-saving strategy and public health promoter against many diseases [15]. In this sense, México, as the site of corn’s origin and domestication, has been responsible for the diversification of this cereal in many forms and colors. México is the country that consumes the second-most corn in LA, below Brazil. However, most of its consumption is for the white (91.5%) or yellow (8%) varieties; colored ones (0.5%) like purple, blue, black, or red are used mostly for ceremonial or ornamental purposes [16]. Mexican black corn has, in its composition, phenolic derivatives from the family of flavonoids known as anthocyanins, responsible for its black-purple color. These are found in the form of cyanidin-3-glucoside (Cy-3-Glu), pelargonidin-3-glucoside (Pg-3-Glu), and peonidin-3-glucoside (Pn-3-Glu). In Mexican blue/purple maize landraces, Cy-Mal-Glu and Cy-diMal-Glu were the major anthocyanins identified. The cyanidin is considered the main aglycone, accounting for 73–75.7% of all the anthocyanins [17]. Promoting its consumption along with other nutritious, easily found local ingredients can be a strategy towards integrating more functional food into day-to-day diets with cultural acceptance and improving the nutritional, metabolic, and general health status of vulnerable populations like children at risk of UN’s deleterious effects [18].

In this order of ideas, this study proposes as a hypothesis that a diet including a black corn chip enriched with other ingredients like a legume (fava bean), a vegetable (nopal cactus), and a cereal (amaranth), even with a globally low protein intake, can improve UN alterations like growth, memory, PA, and energy metabolism parameters, using local ingredients.

## 2. Materials and Methods

### 2.1. Black Corn Samples and Nixtamalization

Black corn was obtained from a local market in Patzcuaro, Michoacán, México. The geographic localization was 19°30′48.44″ N, 101°36′33.08″ W. This corn corresponds to the *Zea mais* L. Chalqueño black variety and was kept under low-humidity conditions, away from direct sunlight at room temperature (18–20 °C); it was collected in September/October 2022. Then, it was shelled to obtain the corn kernels. A solar stove was used to nixtamalize the corn. Nixtamalization is an ancestral precooking method that uses limewater to soften the corn dough and make it more flexible; it also increases the amount of calcium and makes proteins and fiber more digestible [19]. For this process, 60 g of lime was diluted in 3 L of water, then this mixture was heated until it reached a temperature of 80 °C, and then the corn kernels were added. It remained at 80 °C for 45 min, and it was removed from solar rays to stop the cooking process. It was left to rest for 18 h until the next day and was washed with clean water several times to eliminate most of the remaining lime. The kernels were left to dry in low-light conditions until they were ground and used [20].

### 2.2. Local Ingredients

The back corn chip was made with local ingredients such as black, nixtamalized corn (75%), fava bean (dried and ground) (12%), amaranth (5.8%), sun-dried cactus (nopal) (1.7%), oil (soy 5%), water, and salt. All ingredients were obtained from local markets, with the exception of the dehydrated cactus which was provided by the Universidad Intercultural Indígena de Michoacán. After the nixtamalization process, the corn and the fresh nopal cactus were dehydrated, ground with a food grinder, and preserved at room temperature (18–20 °C) in opaque plastic containers away from any source of light and heat until use (after less than 2w).

**Figure 1:**
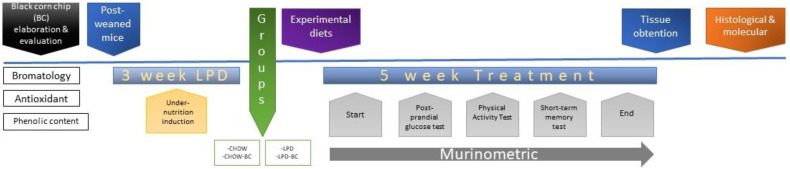
Timeline and experimental execution of the study. First, the ingredients were obtained to elaborate the enriched black corn chip (BC). Then, UN was induced in post-weaned mice (C57Bl/6j), and the experimental diets were started. During the 5w treatment, murinometric data (weekly) were obtained; glucose, physical activity, and short-term memory were tested, and tissue obtention was performed. Finally, all histological and molecular probes were conducted.

A graphical procedure of the experimental execution is shown in Figure 1.

### 2.3. Elaboration of Black Corn Chip (BC)

The ingredients, namely black nixtamalized corn, dried nopal cactus, fava bean, and amaranth were weighed and ground to obtain a homogeneous powder. Then, warm water, vegetable oil (soy), and salt were added. The ingredients were kneaded until a soft dough was formed which was then left to rest for 30 min, protected from light. After that, regular tortillas were formed with a press and cooked over a hot pan; they were cut into eighths and baked for 20 min at 100 °C.

### 2.4. Bromatological Analysis

The bromatological analysis was performed at the Multidisciplinary Laboratory of the Instituto Tecnológico Superior de Tacámbaro (Tacámbaro, México). A sample of the chips was ground until a fine powder was obtained to perform all the analyses in triplicate. The following determinations were analyzed: dry matter [21] (DM method by NMX-F-083-1986), crude protein, crude fiber, ethereal extract (fat), mineral matter from AOAC Official Methods of Analysis [22], and by difference, the carbohydrate content (CH) was calculated.

### 2.5. Antioxidant Capacity

#### 2.5.1. Phenolic Compound Extraction

In order to obtain black corn chip phenolic extracts, chips were ground to a fine powder. Then, 2 g of chip powder was mixed with 20 mL of hexane and then with 15 mL of methanol/water (80:20 *v*/*v*) [23]. The mixture rested for 24 h at 45 °C in dark conditions, and then it was centrifugated at 3600 rpm for 15 min [24]. The supernatant was removed, centrifugation was repeated twice, and the supernatant was collected. It was then evaporated with a Laborota 4000-efficient Heidolph rotary evaporator (Schwabach, Germany) at 50 °C (120 rpm over 10–12 min) and recovered with 5 mL of methanol/water (80:20, *v*/*v*). Finally, it was filtered with cellulose membranes (0.45 μm) and stored at −18 °C until use [25]. Total phenol content was determined by the CIDAM laboratories (cidam.org, result report No. POSC24-0518-1 and 2, Morelia, México) using the Folin–Ciocalteu method. As a control sample, a commercial white corn baked chip was used. Samples were taken in triplicate.

#### 2.5.2. Antioxidant Capacity by DPPH

The DPPH (2,2-Diphenyl-1-picrylhydrazyl) assay is an easy and effective method for evaluating the antioxidant capacity [26] in foods. This method is based on the capacity of radical DPPH (Sigma Aldrich™, Burlington, MA, USA) to change from violet to pale yellow in the presence of antioxidant molecules via a hydrogen atom transfer mechanism. The remaining violet color from DPPH is measured in a UV-VIS spectrophotometer between 512 and 520 nm wavelength [27]. For this assay, 2 mg of DPPH (Sigma Aldrich™, Burlington, MA, USA) was diluted in 100 mL of pure methanol (Sigma Aldrich™, Burlington, MA, USA). Quercetin (Tocris Bioscience, Bristol, UK) was used as a standard to make a control curve at 0, 12.5, 25, 50, and 100 μg/mL. Then, 100 μL of the sample was mixed with 900 μL of DPPH solution and incubated in dark conditions for 30 min. A spectrophotometer was used to obtain the absorbance at a wavelength of 517 nm. To calculate the DPPH-scavenging rate, the following formula was used: DPPH clearance rate = (Ap − As)/Ap × 100, where Ap is the sample’s absorbance (517 nm) and As is the reference absorbance.

### 2.6. Animal Model

Twenty-four C57Bl/6j post-weaned male mice of 3w of age were obtained from a certificate vivarium (UNAM, Juriquilla, Querétaro, México). They were kept in controlled conditions of 12 h/12 h light/dark at a room temperature of 22 ± 2 °C and housed in acrylic cages that contained 3 animals each, with access to water and food ad libitum as indicated in the NIH Guide for the Care and Use of Laboratory Animals and the National Normativity (NOM-062-ZOO-1999). Ethical approval was obtained from the Bioethics Committee of the Instituto Tecnológico Superior de Tacámbaro (ITS T-2023-03). After 72 h of adaptation, the animals’ diet was changed to two types (*n* = 12 each) CHOW (standard diet) or LPD (low-protein diet, −50% vs. CHOW), both diets were made following the indications of Reeves et al. (1993) [28] and Salameh et al. (2019) [29], all diets were isocaloric. An effort was made to minimize discomfort or any kind of pain for the wellness of the animals.

#### Experimental Groups

After 3w of LPD consumption, the undernutrition model was established (body weight vs. CHOW *p* = 0.002). Once the UN model was established, the animals were separated into 4 experimental groups depending on their type of diet: CHOW (standard diet with 14.58% protein intake), CHOW-BC (black corn chip), LPD (low-protein diet with 6.85%), and LPD-BC. The black corn chip dose was determined by the dose of anthocyanins indicated for mice, namely 10 mg/kg of body weight [30], and the quantity of anthocyanins reported in Mexican black corn (93.2/100 g of sample) [31]. So, it was established as 17% of the total diet. All diets had the same amount of kcal/g (3.8 kcal/g). Animals received the type of diet assigned to each group (*n* = 6) for 5 weeks. Table 1 shows the composition of each diet. Appendix A makes a note about preparation and food consumption. To assess the growth of the animals after a 5w treatment, the following murinometric parameters were measured weekly: body weight (g), tail length (cm, from base to tip), and head width (cm, from ear to ear). An electronic balance (Bonvoisin™, Seattle, WA, USA), a fiberglass measuring tape, and a Vernier were used.

### 2.7. Glucose Postprandial Test

UN is related to future metabolic-related diseases (obesity, diabetes, cardiovascular disease, etc.), so to evaluate glucose metabolism, mice were left in a 4 h fast during the dark cycle (when the majority of food consumption happens), glucose was measured with a glucometer (AccuCheck Active, Roche, Basel, Switzerland, purchased from a local Drugstore) from a drop of tail blood, and food was regularly returned to be eaten; 2 h later, glucose was once again measured (American Diabetes Association, Arlington, VA, USA).

### 2.8. Physical Activity Test

To evaluate if the animals’ physical activity, one of the parameters of the expense of energy balance, was affected by the undernutrition induced by the treatment with the black corn chip, an open field test was performed. For this test, animals were left in an evaluation room for 30 min to habituate them to their dark cycle (when they are more active). Then, they were placed in the middle of the open field maze (box of 1 m × 1 m × 35 cm) for 5 min and video-recorded. The Anymaze™ (Wood Dale, IL, USA) program was used to obtain physical activity parameters such as main speed (m/s) and time of mobilization (s). Analyses were performed blindly.

### 2.9. Short-Term Memory Test

To determine if animals had an improvement in their memory, which is important for learning processes and represents one of the most important targets affected by UN, the Novel Object Recognition test was used. It consisted of a first phase in which the animals were left in the open field maze (empty) to familiarize themselves with it (5 min); 24 h later, the animals were left in the open field with two identical objects for 10 min, and 2 h later, they were returned to the open field maze where one of the previous objects was left and a new one was introduced. This test uses the natural curiosity of rodents about new objects. The time of exploration and the % of discrimination of the new object were calculated from this test. The objects used were two stacked tins/cans of tuna and cubes made from plastic blocks, all of which could be climbed by the animals (showing an increased interest). The objects and the open field maze were cleaned with a solution of 70% alcohol between animals. The videos were analyzed blindly; when the animal approached within 1 cm or less of the object with its nose with active vibrissae moving, it was considered a positive exploration [32]. 

### 2.10. Tissue Obtaining and Evaluations

Once the treatment ended, overnight-fasted animals were euthanized by decapitation. The brain was immediately weighed, and the hypothalamus and the white and brown adipose tissues were dissected for molecular probes, which were kept at −80 °C until probes were performed. The liver and kidneys were extracted for histological observation. The length of the right femur was measured as an indicator of body length [33].

### 2.11. Histological Images

The liver and kidneys were put in PFA (paraformaldehyde solution in PBS, pH 6.9 at 4 °C); 48 h later, they were moved to a 10% sucrose solution, and 24 h later, to a 30% sucrose solution. Samples were frozen and then cut into 12 μm thick slices using a cryostat (Leica, Wetzlar, Germany). The hematoxylin–eosin staining method was carried out, and the samples were fixed with synthetic resin to be observed at 10× using an Axioskop microscope 40 (Zeiss, Oberkochen, Germany) with an AxioCam ^MR^ camera linked to Zeiss 2.0 software (Zeiss, Oberkochen, Germany).

### 2.12. mRNA Expression of Energy Balance and Inflammation

#### 2.12.1. RNA Extraction

Total RNA extraction from hypothalamic, epididymal WAT, and BAT was performed using the Trizol–Chloroform protocol as described by the manufacturer, and then the RNA integrity and purity were analyzed using a NanoDrop One/OneC UV–Vis microvolume spectrophotometer (Thermo Scientific, Vacaville, CA, USA).

#### 2.12.2. cDNA Synthesis

First, 2 μg of total RNA was mixed with 1 μL oligo dT (PROMEGA, Madison, WI, USA) and 4 μL milli-Q water in RNAse-free tubes. The mix was incubated for 5 min at 70 °C before the addition of 5 μL of M-MLV buffer 5× (PROMEGA), 1 μL of dNTP mix 10 mM (PROMEGA), and 0.5 μL of RNAsin ribonuclease inhibitor 40 U/μL (PROMEGA). Afterward, 0.2 μL of M−MLV RT 200 U (PROMEGA) was added, incubated for 60 min at 37 °C, and heat-inactivated at 70 °C for 15 min. Finally, the purity and amount of the cDNA were verified by a NanoDrop One/OneC UV–Vis microvolume spectrophotometer (Thermo Scientific, USA).

#### 2.12.3. qPCR

For the qPCR procedure 300 ng of cDNA, 5 μL of Veriquest Fast Sybr Green Master mix (USB Affymetrix Inc., Cleveland, OH, USA), 0.9 μL of direct oligo (10 pM/μL), 0.9 μL of reverse oligo (10 pM/μL), and 0.2 μL of molecular-biology-grade water were used. A final volume of 10 μL per reaction was obtained, which was subjected to reaction in a real-time thermocycler (Bio Rad CFX96, Hercules, CA, USA). The PCR parameters were as follows: the initial denaturation temperature was 95 °C for 5 min, followed by 40 cycles with 15 s denaturation at 94 °C, 30 s alignment at 60 °C, and a 30 s extension at 72 °C followed by 7 min at 72 °C and cooling to 4 °C. The genes analyzed were *Adrb1*, *Adrb2*, and *Adrb3* (WAT and BAT); *Dio2* (WAT, BAT, and hypothalamus); and *OSTa*, *TGR5*, *TNFa*, *Il-1B*, *AGRP*, *NPY*, and *POMC* (hypothalamus). Primer sequences are reported in Table S1. Nono was used as a reference gene. Data were normalized and analyzed by the delta-delta CT method [34], and the mRNA expression levels are expressed as the fold changes from the reference group as indicated by Velázquez-González et al. (2023) [35].

### 2.13. Statistical Analysis

Statistical analyses were performed by means of the GraphPad Prism software, version 8.0.2 (GraphPad Software Inc., San Diego, CA, USA). Data are presented as mean values ± S.E.M (standard error of the mean). A comparison of the experimental data obtained from the different groups was made by a one-way analysis of variance (ANOVA), followed by the Bonferroni post hoc test, and a comparison of the results obtained from the control specimens and the animals treated with the BC was performed by means of a Student’s *t*-test; *p* ≤ 0.05 was considered statistically significant.

## 3. Results

### 3.1. Bromatological Composition

To determine the nutrient content of the BC, a bromatological analysis was performed comparing it with a commercial baked white corn chip. The results are shown in Table 2. These data allowed us to adjust the experimental diets (CHOW, CHOW-BC, LPD, and LPD-BC) in order to have the same macronutrient composition and calories regardless of whether they included the BC or not. The composition showed significant differences in crude protein, ethereal extract, and carbohydrate content. This was expected as their ingredient composition is different; the BC presented a higher protein content due to the addition of the fava bean which, combined with corn, offers all the essential amino acids in comparison with the regular baked chip made only with corn. The BC had a higher lipid content and lower carbohydrate content than the commercial chip.

### 3.2. Phenolic Content and Antioxidant Capacity of Black Corn Chip

**Figure 2:**
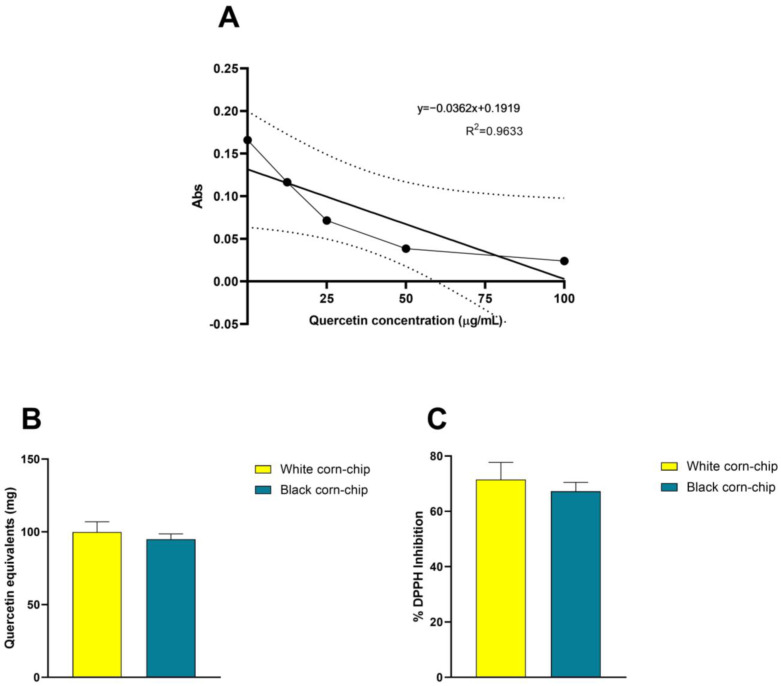
Antioxidant capacity of BC vs. white corn baked chip. DPPH assay was performed using quercetin as control antioxidant. (**A**) The absorbance at 517 nm standard curve of quercetin (μg/mL), lines correspond to linear regression of the curve and error (pointed lines), equation and the R^2^ are included; (**B**) the equivalents of quercetin and (**C**) the % of DPPH inhibition of baked white and black corn chips. Samples were analyzed in triplicate.

For the BC and a commercial baked white corn chip, the total phenolic content was determined by the Folin–Ciocalteu method, and the antioxidant capacity was determined by the DPPH method. The results are shown in Figure 2A–C. It is remarkable how they did not present differences between each other in antioxidant capacity; however, the phenolic content was significantly higher in the BC (* *p* < 0.05). This is an indication of the presence of other antioxidant molecules in the commercial white chip. 

### 3.3. Undernutrition Induction

**Figure 3:**
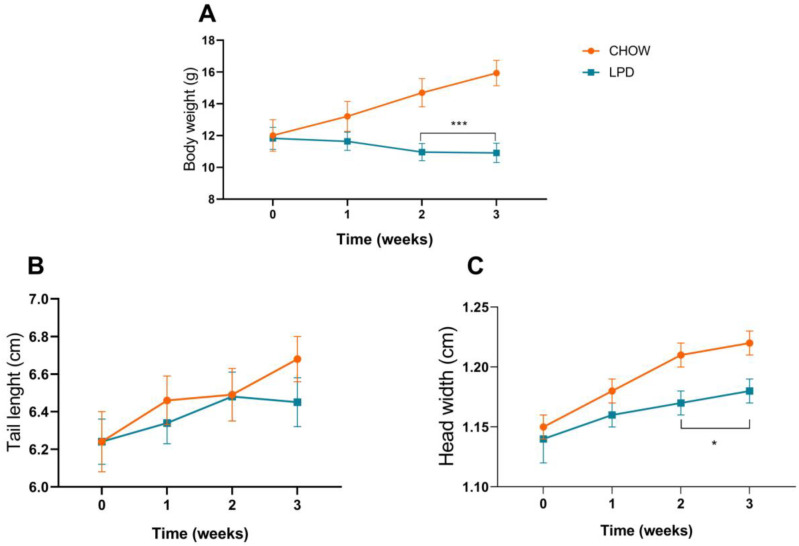
Induction of undernutrition (UN) in post-weaned 3wo mice. Mice received either a CHOW diet or a low-protein diet (50%) over 3 weeks; both diets were isocaloric. Protein restriction significantly affected the (**A**) body weight gain and (**C**) head width; no significant changes were registered during tail length measurement (**B**). A multiple t test was applied; * *p* ≤ 0.05, *** *p* = 0.0001 (*n* = 12 for each group).

Three-week-old male C57Bl/6j mice (post-weaned) received either a standard diet (CHOW) or an isocaloric low-protein diet (LPD −50% protein diet). After 3w of induction, the mice were considered undernourished, with a significant decrease in body weight (BW) after the 2nd week (*p* = 0.002) which continued to worsen over time. This work reached a successful induction of acute UN in male mice weaned only with an LPD (isocaloric but 50% of regular protein input) as evidenced by the decrease in and loss of body weight gain (final %BW change of CHOW: 136.18 ± 5.3 vs. LPD: 92.48 ± 1.38 **** *n* = 12). Other parameters considered to assess UN effects were tail length (final *p* = 0.21), which was not affected in this period of time, and head width, which was affected in this induction, showing how sensitive the central nervous system is to restrictive nutrient intake (at the 2nd and 3rd weeks, *p* = 0.017) Figure 3.

#### 3.3.1. Black Corn Chip Treatment’s Effects on Murinometric Parameters

**Figure 4:**
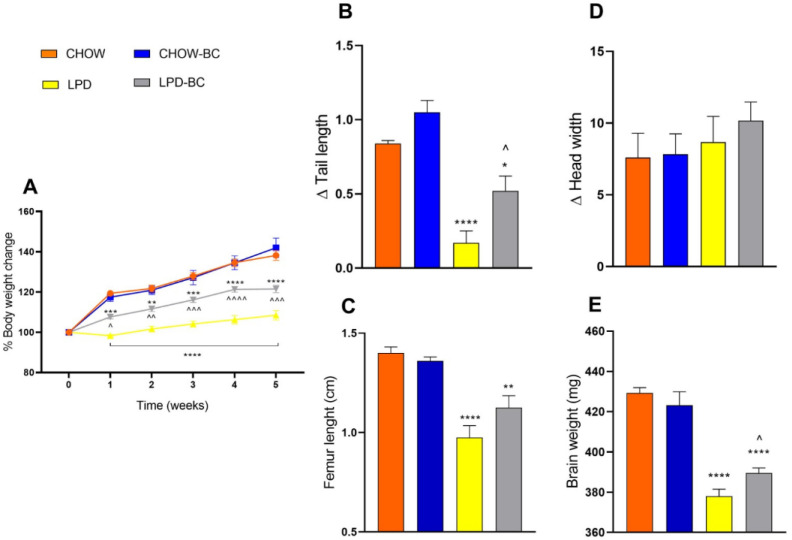
Treatment of a UN model with a 17% addition of a black corn chip. The graph shows the % of body weight gain (**A**), the length increase evidenced by a significant change in tail length, and less difference in femur longitudinal growth vs. CHOW (**B**,**C**); finally, murinometric data showed a tendency for head width to increase and a statistically significant improvement in brain weight (**D**,**E**). Data were analyzed by one-way ANOVA, with a Bonferroni post hoc test, (*n* = 6); * *p* ≤ 0.05, ** ≤0.009, *** ≤0.0001, **** ≤0.00001 vs. CHOW, ^ *p* ≤ 0.05, ^^ ≤0.009, ^^^ ≤0.0001, ^^^^ ≤0.00001 vs. LPD.

Once induction was reached, treatment with the BC started and was continued for over 5 weeks. Data of murinometric parameters under BC treatment (Figure 4A–E) were united to appreciate global results. Once more, the body weight, tail length, and head width were considered, showing significant improvement.

#### 3.3.2. Body Weight

Once undernutrition was established, mice groups began with a 5w BC treatment (added at 17% of the total diet). Murinometric data showed a significant increase in the percentage of body weight gained with BC treatment (*p* = 0.01 to 0.0002 since 1st week vs. LPD) (Figure 4A) Even though all the diets were isocaloric and the BC treatment maintained a low protein intake, body weight improvement was independent of calorie and protein intake.

#### 3.3.3. Body Length

Tail length, as a reflection of longitudinal mice size, also showed a significant increase with BC treatment (*p* = 0.02 vs. LPD). Femoral post mortem length was also measured to evaluate longitudinal gain, showing a tendency to increase with BC treatment (*p* = 0.11 vs. LPD) (Figure 4B,C).

#### 3.3.4. Cephalic Development

To assess cephalic growth as an indicator of cognitive development, head width and cerebral weight were considered in this study. Data showed only a tendency of an increase in head width with BC treatment and a significant augmentation of the brain mass of the UN mice treated with the BC (*p* = 0.02 vs. LPD) (Figure 4D,E).

### 3.4. Glucose Postprandial Test

**Figure 5:**
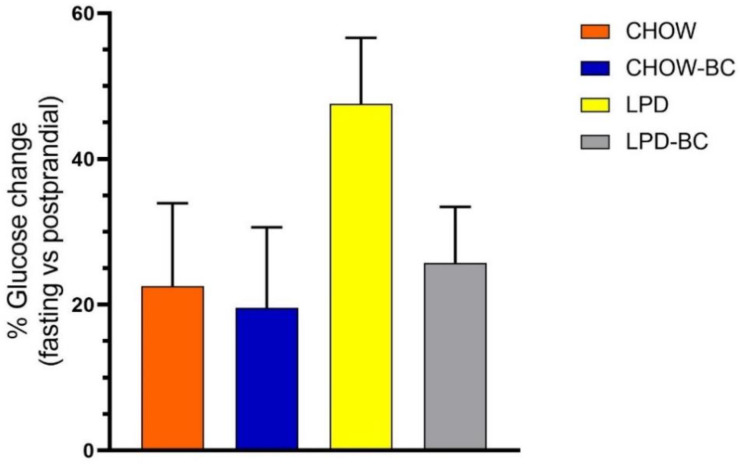
Postprandial glucose change. The graph shows the % of change between the basal level and 2 h post-meal. CHOW, CHOW-BC, and LPD-BC had a similar augmentation; the glucose levels of the LPD group had a tendency to increase with no significant outcome (one-way ANOVA, *p* = 0.21, *n* = 5).

Glucose metabolism is an important indicator of the general nutrient and energy status of the organism. It reflects the correct function of muscle, liver, adipose tissue, and brain. However, the postprandial glucose evaluation in this study (Figure 5) did not show any significant differences between groups of treatment. It is notable that there was a tendency for the LPD group to have a higher increase in glucose after meal consumption, while the LPD-BC group had a similar response to that of the CHOW group (normal).

### 3.5. Physical Activity

**Figure 6:**
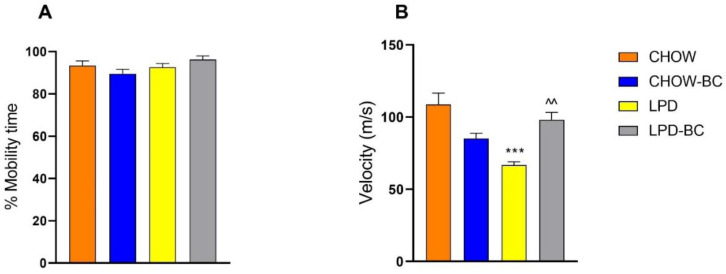
Physical parameters like (**A**) mobility time and (**B**) velocity showed an equal time of mobility with all groups being very active, but the velocity results of LPD were significantly lower compared to those of CHOW (*** *p* ≤ 0.0001), while LPD-BC’s results were significantly higher (^^ *p* = 0.004) vs. LPD.

As part of the energy balance between income (nutrients) and outcome (basal metabolism, thermogenesis, and physical activity) (Figure 6), mobility time (Figure 6A) and velocity (Figure 6B) were measured to establish how active the mice were and how undernutrition and treatment affected these parameters. Mobility time did not show alterations caused by the nutritional status; however, velocity did, presenting a significant increase in UN animals under BC treatment (^^ *p* = 0.004 vs. LPD).

### 3.6. Short-Term Memory

**Figure 7:**
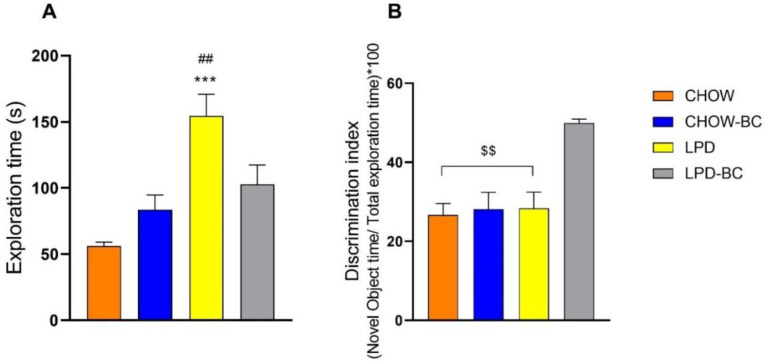
Short-term memory test evaluated by Novel Object Recognition. The total exploration time (**A**) shows an increased interest of the LPD group in the objects (indiscriminately) (*** *p* ≤ 0.0005 vs. CHOW, ^##^ *p* ≤ 0.009 vs. CHOW-BC), but, when identifying the novel object, the LPD-BC group had higher discrimination index (**B**) (^$$^ *p* ≤ 0.009).

One of the most concerning alterations of undernutrition is in learning ability; memory is a vital part of the consolidation of this process. Low protein intake is a well-established factor that impairs memory and learning in the early stages of life. Data showed (Figure 7) a significant improvement in short-term memory with BC treatment punctually on the discrimination index (capacity of recognizing a new object vs. a familiar one) (Figure 7B). In parallel, the LPD group showed more curiosity about the objects, which was translated as exploration time (Figure 7A).

### 3.7. Histological Analysis

**Figure 8:**
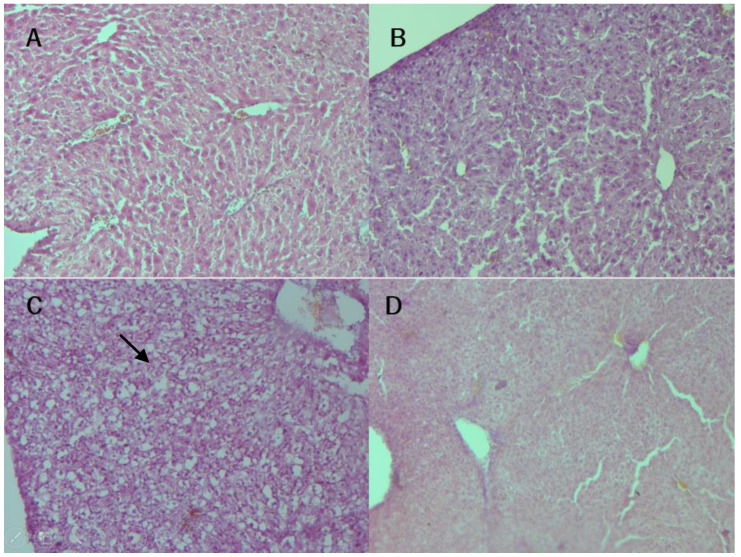

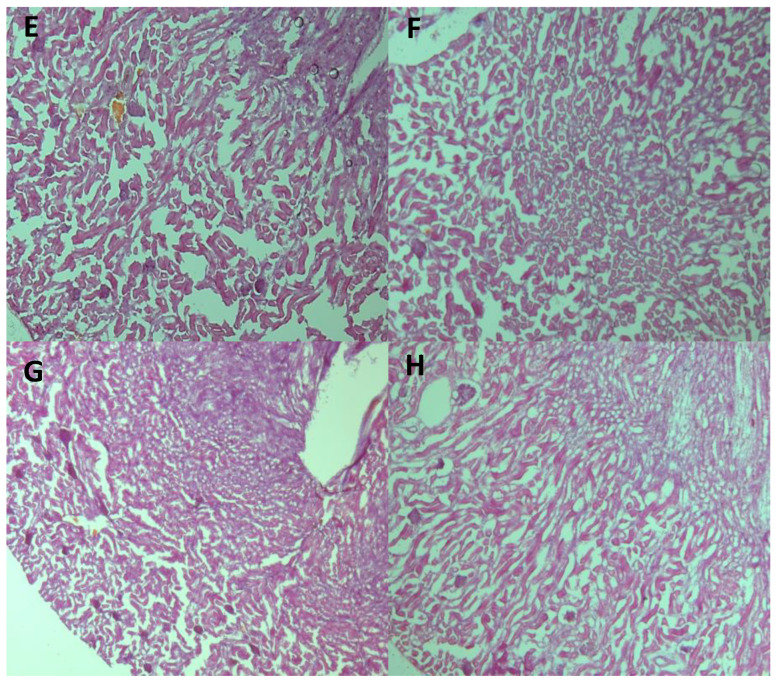
Micrograph of 12 μm cuts of liver (**A**–**D**) and kidney (**E**–**H**), (**A**,**E**) from CHOW, (**B**,**F**) from CHOW-BC, (**C**,**G**) from LPD, and (**D**,**H**) from LPD-BC groups. In the (**C**) (LPD) liver cut, the black arrow indicates the presence of white circles, a distinctive trait of UN steatosis.

In order to rule out any alterations (toxicity, steatosis, inflammation, or necrosis) caused by BC treatment, a histological analysis was carried out. A qualitative analysis (image comparison Figure 8A–D) showed the presence of steatosis in the UN liver evidenced by white circular spaces [36,37], but the alteration did not appear with BC treatment. Nephron morphology (Figure 8E–H) did not present any alterations among the different treatments; under this type of staining, toxicity damage generates red protein plaques [38], which did not show up.

### 3.8. Gene Expression

**Figure 9:**
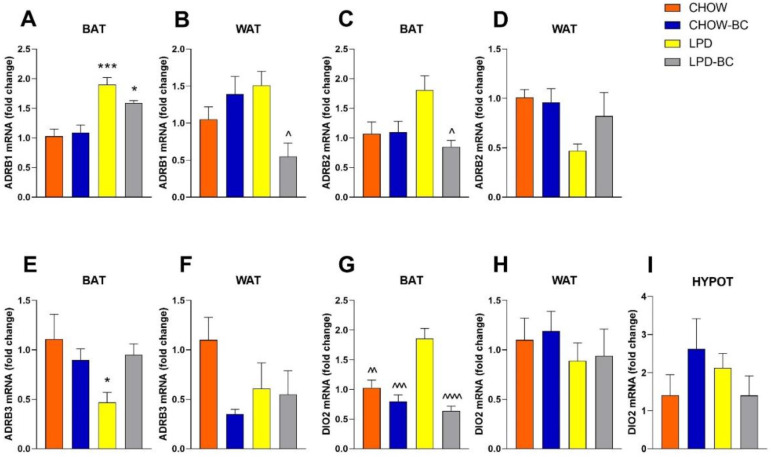
ADRB1, ADRB2, ADRB3, and DIO2 gene expression in BAT, WAT, and hypothalamus. (**A**,**B**) ADRB1 is involved in thermogenesis in brown adipose tissue (BAT). UN promotes the upregulation of thermogenesis, which is also elevated in LPD-BC treatment. It is also responsible for the increase in heat production of browning white adipose tissue (WAT). LPD-BC treatment significantly lowered ADRB1 in WAT. ADRB2 and ADRB3 are key regulators of energy balance, regulating lipolysis and thermogenesis; in BAT, both suffered normalization with BC treatment in the UN model (**C**,**E**); in WAT (**D**), there is a tendency towards the normalization of levels of ADRB2, and (**F**) shows a tendency towards the downregulation of ADRB3 in CHOW-BC and UN groups. (**G**–**I**) DIO2 is implicated in the activation of thyroid hormones that regulate metabolism; UN shows a significantly increased level in BAT (**G**), consistent with the thermogenic production of this vulnerable group. However, BC treatment normalized the BAT-DIO2 levels. Data analyzed by one-way ANOVA with a Bonferroni post hoc test, * *p* ≤ 0.05, *** ≤0.0001, vs. CHOW and ^ *p* ≤ 0.05, ^^ ≤0.009, ^^^ ≤0.0001, ^^^^ ≤0.00001 vs. LPD; the number of marks represents the number of zeros after the decimal point, indicating more significance, *n* = 4–6.

**Figure 10:**
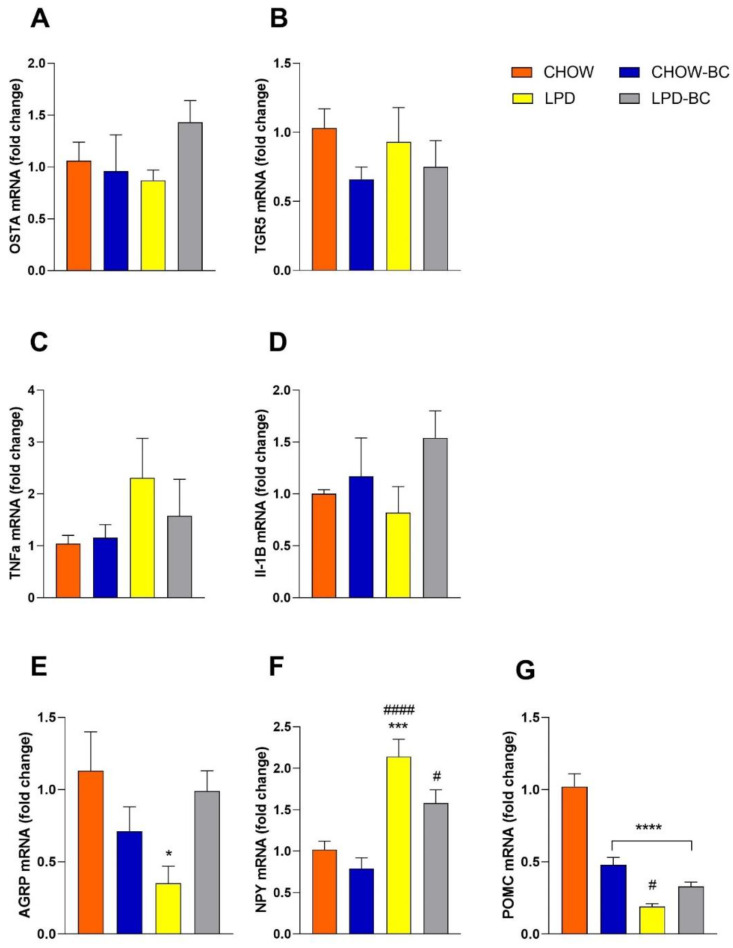
Gene expression involved in (**A**,**B**) bile acid transporter (OSTA) and receptor (TGR5) implicated in the regulation of metabolism; (**C**,**D**) TNFa and Il-1B are proinflammatory cytokines, and (**E**–**G**) are genes from melanocortin system (energy balance) expressed in the hypothalamus. Data analyzed by one-way ANOVA with a Bonferroni post hoc test, * *p* ≤ 0.05, *** *p* ≤ 0.0001 and **** ≤0.00001 vs. CHOW, ^#^ *p* ≤ 0.05 and ^####^ *p* ≤ 0.00001 vs. CHOW-BC; the number of marks represents the number of zeros after the decimal point, indicating more significance, *n* = 4–6.

The expression of genes implicated in thermogenesis (AGRP1, AGRP2, AGRP3, and DIO2) in adipose tissue (brown (BAT) and white (WAT)) and the hypothalamus (DIO2) (Figure 9) and the expression of genes implicated in the activation of metabolism (OSTa and TGR5), inflammation (TNFa and Il-1β), and the melanocortin system (AGRP, NPY, and POMC) in the hypothalamus (Figure 10) were measured. This gene expression analysis showed the normalization of AGRP/NPY with BC treatment and a lowering effect of thermogenesis/browning WAT (Figure 9A–C). The DIO2 gene is implicated in metabolism that is regulated by thyroid hormone, responsible for activating T4 into T3, BC treatment significantly decreased its expression in BAT (Figure 9C); neither WAT nor the hypothalamus showed a difference between groups. This explains how an adjustment in metabolism towards normality was driven by BC treatment, with a tendency to save energy.

Regarding OSTa and TGR5 (Figure 10A,B), studies highlight their implication in energy metabolism regulation as a transporter and receptor of bile acids, respectively. Together, they modulate glucose, triglycerides, cholesterol, and energy homeostasis [39]. As previously mentioned, UN, oxidative stress, and inflammation co-exist very closely, so to evaluate hypothalamic inflammation and its possible disruption in the UN condition and its treatment with the BC, TNFa and Il-1β gene expression was measured; BC treatment presented a tendency to downregulate TNFa in UN animals but, on the other hand, a tendency to increase IL-1β (Figure 10C,D). Finally, the expression of key melanocortin system genes (AGRP, NPY, and POMC) showed a normalization in AGRP and NPY levels of UN animals with BC treatment (significantly). AGRP and NPY increase when low levels of glucose, insulin, and leptin occur with fasting [40] and promote food intake; however, in low-protein conditions, an interest in seeking protein-rich food increases and an active rejection of low-protein food appear [41]. POMC was significantly lowered in UN groups, but also in the CHOW-BC group (Figure 10E), probably as a form of protection from satiety.

## 4. Discussion

The determination of the content of nutrients and antioxidant capacity of the BC showed an improvement in the quality of nutrients in comparison with the commercial product (only made with regular white corn), as it has more protein (~2.8 g/100 g), but also, the protein quality is improved as it combines the amino acids from the corn (a cereal) with the amino acids from the fava bean (a legume), which combined with amaranth, provide a profile of essential amino acids similar to those found in animal protein [42]. This is a sustainable and economical way of replacing animal protein as legumes offer the essential amino acids that cereals lack and consumers do not notice their presence in the products fortified with them [43,44]. Another nutritional improvement was the significant decrease in carbohydrate content and an increase in general mineral input provided along with the other ingredients. However, in terms of antioxidant capacity, no apparent improvement was shown. A possible explanation for this could be the addition of antioxidants to the commercial product to avoid rancidness (to prolong the product’s shelf life); generally, tocopherols or butylhydroxytoluene (BHT) are used. Studies highlight the natural presence of tocopherols in corn and the variation that occurs along its maturation process [45]. However, both chips had high antioxidant capacity according to several standard antioxidants published by Albanese et al. (2019) [46], the only difference being that one has anthocyanins and the other does not; approximately 69.9 ± 1.1 mg of cyanidin 3 glucoside/100 g is present in the content of the black corn chip sample [31].

Once the BC was enriched, the next step in elucidating its effects on nutritional status was to probe it in vivo using a murine UN model. This probe allowed us to measure the response of a complex organism and the systemic metabolism of the BC components. The UN model with low protein intake, according to Salameh et al. (2019) [29], is a model that is representative of the growth of children of similar low-income countries who present weight loss. Tail length, the murinometric parameter that reflects animal body length, did not show a significant difference, but other works evaluating UN and tail length did find differences; we hypothesize that more time of induction was probably needed for tail length to be affected in these animals. This effect was reached after the 5w lapse time of treatment (Figure 4B). Head width was taken as a murinometric parameter of cerebral development. In children, growth evaluation takes the circumference of the head from the day of birth until children reach 36 months old as a base. This parameter was significantly affected after 2 weeks of LPD (*p* = 0.0009 vs. CHOW), suggesting poor cerebral development and possible compromise of cognitive capacities [47,48]. This response is consistent with the fact that when essential nutrients like the amino acids leucine and tryptophan (deficit of protein), oxygen, energy, and growth factors are scarce, mTORC1 is inactivated, resulting in the inhibition of lipid and protein synthesis and the activation of autophagy. This principally affects muscle growth, bone length, myelination of the nervous system (central and peripheral), organ size, and iron metabolism [49].

After induction, the treatment with the BC was applied to UN mice and to one control CHOW group to evaluate the response in a healthy normal-weight organism and to rule out possible obesogenic (above-normal gain in body weight) effects on normal-weight individuals. The 5w BC treatment showed a significant improvement in the murinometric parameters of UN mice, as evidenced by BW gain (*p* = 0.0002 vs. LPD), tail length (*p* = 0.02 vs. LPD), improved femur length (*p* < 0.0001 for LPD vs. 0.003 for LPD-BC vs. CHOW), a tendency towards the normalization of head width (*p* = 0.196 vs. CHOW), and a significant enhancement in brain weight (*p* = 0.01 vs. LPD). On the other hand, in the CHOW-BC group, the normality of the aforementioned murinometric parameters was maintained, ruling out the presence of any type of obesogenic effect on the BC normal-weight subjects.

When adequate nutritional supply is compromised, mainly during the first five years of life, several important factors including cognitive development suffer [50]. Early stages of life and nutritional status are crucial factors in consolidating learning and memory [51]. In this sense, this study aimed to evaluate short-term memory by applying the Novel Object Recognition test. The results showed that the BC treatment applied to a UN model significantly increased (*p* = 0.002 vs. LPD) the % of discrimination time of the novel object, which means that the animal remembered that the novel object was not there 2 h earlier, showing greater interest in it. This is consistent with the data of Ip and collaborators (2017) [52], who pointed out the importance of the supplementation of food with iron, zinc, calcium, and protein for improved cognitive effects in children from different developing countries. Food supplementation with polyphenols and fatty acids has shown a significant improvement in memory and learning in humans [53]. Colored corn and the enrichment of food based on this ingredient are of wide interest in many fields of science and technology. Studies have shown its antioxidant, anti-inflammatory, and anticarcinogenic capacities, as well as its many effects on metabolic dysregulations like obesity, diabetes, and hypertension. These benefits are attributed to its rich composition, which includes phenolic compounds, anthocyanins, fibers, phytosterols, ferulic acid, phospholipids, fatty acids, et cetera [18,24,30,54].

Another parameter of interest in this test was the total time of exploration, that is, the time the mice spent smelling the objects in the arena. These data showed a significant increase in the exploring interest of the LPD group in comparison to the CHOW group. However, there was only a tendency of an increase vs. the LPD-BC group; these data suggest that UN mice tended to be more curious but did not recognize the fact that a novel object was there. This phenomenon is explained by the increased foraging conduct, that is, the active seeking of food, that individuals with UN present [41].

A postprandial glucose test allows the evaluation of the response of an organism in the management of glucose peaks after a meal. A postprandial challenge is altered in undernourished conditions, which means a lower quantity of muscle and adipose tissues (insulin-responsive tissues that are important in the glucose absorption process). The postprandial glucose findings in this study showed a tendency towards a lower response in the UN group without BC treatment and are consistent with studies that relate early nutritional deficiency with metabolic adult problems like T2 diabetes and obesity due to a reduction in insulin secretion [55]. Neither hepatic nor renal tissue damage was found with treatment; furthermore, the treatment with the BC avoided steatosis related to undernutrition [36,37]. Gene expression showed that the main effects of BC treatment in a UN model (even with low sustained protein intake) were recovery from altered thermogenesis and a normalized energy balance related to orexigenic response. Regardless of the expected explanation linked to antioxidant activity, the BC did not present any differences compared to a similar commercial product made with white corn, so all its evident beneficial outcomes are probably linked to other functional properties; we could hypothesize that micronutrient enrichment or biomolecule functionality is an alternative to antioxidant capacity only. These results agree with Lao et al. (2017) [30] about the lack of identification of the most effective functional phenolic component and the suggestion of it being a synergistic interaction between several molecules, like vitamin C (present in nopal cactus), which increases in presence in the liver and adrenals if it is consumed with anthocyanins [56]. Therefore, these molecules contribute to the observed beneficial effects and keep their functionality regardless of the cooking methods.

## 5. Conclusions

The consumption of a functional baked black corn chip (BC) showed efficacy in reversing some of the most concerning aspects of undernutrition. It showed efficacy in recovering body weight independently from the quantity of protein and calories and a tendency to promote length and neurocognitive recovery and prevent undernutrition-related hepatic steatosis. Overall, gene expression showed that BC treatment decreased energy expenditure from thermogenesis and promoted a normalization in food intake modulated by neuropeptides NPY/AGRP. We hypothesize that anthocyanins present in the BC can act synergistically with other nutritious and non-nutritious components in metabolically important tissues and their central regulators. Other biomolecules found in the BC ingredients are polyphenols (like quercetin and kaempferol from nopal cactus), minerals (calcium, magnesium, potassium, zinc), and vitamins (retinol, folic acid, vitamins C and E), which modulate other aspects like the microbiome, growth hormones, regulatory peptides, and the endogenous antioxidant system, to mention a few. Some of the limitations this study presented related to the quantification of the food consumed; those data could give information regarding appetite/satiety and food efficiency (the capacity to transform kcal consumed into body mass). In addition, this study could have benefited from anthocyanin quantification and characterization; as the chip is a mixture of several ingredients, the total and type of polyphenols would have been enriching to the study. So, further studies are needed to elucidate the possible mechanisms involved in the improvement effects on the UN mouse model, such as food consumption, anthocyanin and polyphenolic characterization of the chip, microbiome information, long-term memory, and learning; in addition, immune probes can be performed. Overall, this study supports the use of the BC as a safe and nutritious alternative to be integrated on a daily basis for children from low-income communities, even with adjustments to the ingredients to make it culturally and economically suitable, all while using an affordable, culturally accepted, and sustainable approach.

## Figures and Tables

**Table 1:** Diet composition of the experimental groups with different protein contents but the same calorie content.

	CHOW	CHOW-BC	LPD	LPD-BC
Corn Starch	621	510.8	710	595
Sucrose	100	100	100	100
Casein	140	123.2	65	48.5
Oil (Soy)	44	11.2	30	2.5
Vitamin Mix	10	10	10	10
Mineral Mix	35	30.8	35	30.8
Celulose	50	45.8	50	45.8
Black Corn Chip	0	170	0	170
Carbohydrate	75.10%	75.10%	84.85%	84.85%
Protein	14.58%	14.58%	6.85%	6.85%
Lipids	10.31%	10.31%	8.30%	8.30%
Kcal/g	3.84	3.84	3.8	3.79

**Table 2:** Nutritional composition of black corn chip (BC) vs. white commercial baked corn chip. Data are expressed in g/100 g of product.

Component	Black Corn Chip	Baked White Corn Chip
Humidity	1.58 ± 0.36	2.32 ± 0.26
Crude protein	9.80 ± 0.18 *	7. 0 ± 0.4
Crude fiber	3.64 ± 0.48	2.13 ± 0.56
Ethereal extract	22.62 ± 2.00 *	19.0 ± 0.27
Mineral matter	2.32 ± 0.02	0.98 ± 0.06
Carbohydrate ^a^	60.04 *	68.57
Total phenols ^b^	0.84 *	0.48

^a^ Carbohydrate content is calculated by the difference of the summation of the other contents. ^b^ Total phenols are expressed on mg equivalents of gallic acid/g sample. Data were compared with *t*-test, * p ≤ 0.05.

## Data Availability

Data described in the manuscript, code book, and analytic code will be made available upon request due to privacy reasons.

## Supplementary Materials

The following supporting information can be downloaded at: https://www.mdpi.com/article/10.3390/nu16132001/s1, Table S1. Primer sequences used for qPCR.

## Appendix A

Food preparation. In order to make a specific composition of the diets, the indications of Reeves et al. (1993) [28] were followed. The final mix had a powder texture, so, to make it more similar to the chow diet, a known amount of water was added, and a pellet was formed and then dehydrated at 60 °C for 12 h to achieve the original weight (pre-hydration). Consumption, however, was hard to follow because mice would turn the pellets into powder again to make a food mixture with bedding and urine, complicating the weighing of the food left after consumption.
