# Supplementary material for: Integrative Approach of Treating Early Undernutrition with an Enriched Black Corn Chip, Study on a Murine Model
Source: Nutrients. 2024 Jun 24;16(13):2001. doi: 10.3390/nu16132001 (PMC11243394; doi:10.3390/nu16132001)
Supplement: Supplementary file 1 [file nutrients-16-02001-s001.zip › nutrients-3019488-supplementary.pdf]

Table S1. Primer sequences used for qPCR.

Gene name	GenBank ID	Forward primer (5'-3')	Reverse primer (3'-5')
Nono	53610	CTG TCT GGT GCA TTC CTG AAC TAT	AGC TCT GAG TTC CCA TG
Adrb1	11554	TTC TCC TAG AGG GCA AAC CTT GT	CAG AGT GAG GTA GAG GAC CCA CA
Adrb2	11555	CTG TGC CTT CGC AGG TCT TC	TCC GTT CTG CCG TTG CTA TT
Adrb3	11556	CGA CAT GTT CCT CCA CAA ATC A	TGG ATT CCT GCT CTC AAA CTA ACC
Dio2 BAT	NM_010050.4	CAG ACT CAC CAG CCC ATG TAA C	CGC ACA CCA GTG AGC TCT GA
Dio 2 HYP	13371	CCA CCT GAC CAC CTT TCA CT	TGG TTC CGG TGC TTC TTA AC
Tgr5	227289	CCT TTC CCT GCT TGC CAA T	CCG GAG TGG CTG CAA CAC
Osta	106407	TTG GAC CCT GGA AGA CAT ACT GTA	CTG ATA AGG CTG AGG GAC AGA AG
Il-1B	16176	GAA GAA GAG CCC ATC CTC TG	TCA TCT CGG AGC CTG TAG TG
Tnfa	21926	GCC TCT TCT CAT TCC TGC TT	TGG GAA CTT CTC ATC CTT TT
Agrp	11604	CTT TGG CGG AGG TGC TAG AT	AGG ACT CGT GCA GCC TTA CAC
Npy	109648	CCC CAG AAC AAG GCT TGA AG	TTG GAA AAG TCG GGA GAA CAA
Pomc	18976	TGG GCG AGC TGA TGA CCT	GCC CAG TGT GAA ATC TGA AAG G
